# High-Cutoff Hemodialysis Therapy for Patients with Light Chain Cast Nephropathy and AKI Requiring Dialysis: CON

**DOI:** 10.34067/KID.0000000000000150

**Published:** 2023-05-22

**Authors:** Nithin Karakala, Luis A. Juncos

**Affiliations:** 1 Department of Medicine, Division of Nephrology, University of Arkansas for Medical Sciences, Little Rock, Arkansas; 2 Department of Medicine, Division of Nephrology, Central Arkansas Veterans Healthcare System, Little Rock, Arkansas

**Keywords:** acute kidney injury and ICU nephrology, acute kidney injury, free light chains, high-cutoff dialysis, multiple myeloma, renal dialysis

Multiple myeloma is a common hematologic malignancy, with a worldwide incidence of 160,000,^1^ of which approximately 35,000 are in the United States It is characterized by uncontrolled clonal proliferation of plasma cells associated with secretion of monoclonal immunoglobulin and/or free light chains (FLCs). Approximately 30% of those diagnosed with multiple myeloma have renal impairment at diagnosis.^2^ Of these, 25% have AKI,^3^ with approximately 10% developing severe AKI.^3^ Although advances in its therapy have improved survival over the past 20 years,^4^ the mortality rates among patients with AKI, particularly myeloma cast-associated AKI (MC-AKI), remains high.^5^ Hence, much research has focused on deciphering the mechanisms of MC-AKI. In this regard, FLC burden plays an important role and correlates with an increased risk of MC-AKI and increased mortality.^6,7^ Thus, strategies that reduce their burden have been proposed. This debate focuses on whether extracorporeal clearance of serum-free light chains (sFLCs), particularly *via* high-cutoff hemodialysis (HCO-HD), should be used as adjunctive therapy in the treatment of MC-AKI (Figure 1).

**Figure 1.**
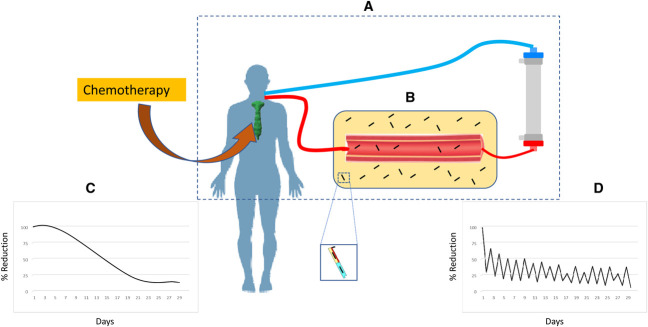
**Free light chain clearance using high-cutoff hemodialysis therapy.** (A) HCO dialysis circuit, (B) distribution of abnormal FLCs (20% intravascular and 80% extravascular), (C) rate of FLC reduction with 10% tumor kill per day* with the use of chemotherapy, and (D) the rate of FLC reduction with a combination of chemotherapy and HCO dialysis*, rapid reduction of FLC from the serum with dialysis, followed by redistribution and rebound increase in serum concentration. FLC, free light chains; HCO, high-cutoff. *Simulation models.

## Rationale for Reducing the FLC Burden in Preventing or Treating MC-AKI

The chief instigator of MC-AKI is the FLC. Approximately 500 mg of polyclonal FLCs are produced per day *via* normal lymphoid metabolism. Approximately 60% are incorporated into intact immunoglobulins, with the excess circulating as sFLCs that are rapidly cleared by the kidney. FLCs are filtered at the glomerulus and then reabsorbed *via* a megalin-cubilin–dependent mechanism in the proximal tubule. During myeloma, the increased production, and consequently filtration, of the monoclonal FLCs leads to their enhanced proximal tubular reabsorption. This, in turn, initiates a cascade of inflammatory processes, including the activation of nuclear factor-_Κ_B, mitogen-activated protein kinase, and extracellular signal-regulated kinase, eventually leading to upregulation of apoptotic pathways in these cells. In addition, the increased FLCs load exceeds the absorptive capacity of the proximal tubule, resulting in increased quantities of FLCs reaching the more distal segments. Here, the third complementarity-determining region (CDR3) of both *Κ*, and *λ* FLCs binds to a nine-amino acid sequence on the uromodulin protein and gets trapped in the gel. The above interaction is influenced by pH, NaCl, and Ca concentration.^8^ The rapid aggregation of these proteins leads to the formation of casts, which subsequently causes intratubular obstruction and tubular atrophy.^9^ These casts, together with the inflammatory milieu caused by the cellular effects of FLCs, can promote rapid tubulointerstitial fibrosis. Consequently, rapid and aggressive depletion of sFLC levels is crucial if we are to mitigate the development of MC-AKI.^10^ The fundamental basis of therapy is plasma cell depletion *via* chemotherapy. However, because the effects of chemotherapy are not always immediate and time is of essence, rapid extracorporeal removal of sFLCs has been used as adjunctive therapy. Hence, the strategy is solid. However, as Winston Churchill once said, “No matter how brilliant the strategy, we must occasionally look at the results.”

## Effectiveness of Extracorporeal FLC Removal on MM-AKI

The first and most commonly used modality for extracorporeal clearance of sFLCs was therapeutic plasma exchange (TPE). It was being used successfully in other paraproteinemias and thus proposed as an effective way to remove FLCs. This was supported by early case reports and series suggesting that it could be effective. A randomized study by Zucchelli *et al.*^11^ also suggested that its use led to better renal recovery and survival. However, this study had various limitations, including small sample size and the use of different therapeutic regimens between the groups. Despite these early positive results, two subsequent randomized trials failed to demonstrate a benefit. Despite the lack of solid supportive data supporting its role in MC-AKI, its use reportedly increased over the past two decades.^12^

A disadvantage of TPE is the indiscriminate removal of most plasma proteins, which renders it less efficient and can contribute to complications because of the loss of essential proteins (*e.g*., clotting factors). Thus, a strategy that more selectively removes proteins in the FLC size range (22.5–45 kD) was devised by performing hemodialysis (HD) using HCO-HD filters, which have molecular mass cutoffs of 45–60 kD. Indeed, the efficacy of this strategy was demonstrated *in vitro* and *in vivo* by Hutchison *et al.*^13^ They achieved a near-absolute reduction of FLCs from the serum *in vitro* and clearance rates ranging from 9 to 30 ml/min in human subjects by using a polyarylethersulfon dialyzer with a molecular cutoff of 45 kD. This prompted two randomized clinical trials examining the benefits of HCO-HD as adjunct to plasma cell-depleting chemotherapy. In the prospective multicenter MYRE trial,^14^ patients with MC-AKI with biopsy-proven cast nephropath were enrolled. Ninety-eight patients who required renal replacement therapy were randomized to receive either standard HD or HCO-HD. Despite the higher sFLC clearance in the HCO-HD group, it did not demonstrate a benefit on the hard outcomes of survival or dialysis independence at 3 months. Intriguingly, dialysis independence at 6 and 12 months was higher in the HCO-HD group, but with a very low fragility index, demonstrating a lack of robustness of these data. The second study, the EuLITE trial,^15^ was a multicenter study across 16 centers in the United Kingdom and Germany, that randomized 90 patients with MM-AKI, with intratubular casts on biopsy to receive either conventional high-flux HD or extended (8 hours) HCO-HD in addition to bortezomib-based chemotherapy. It also failed to detect any clinical benefit of HCO-HD over conventional HD, including on renal recovery or dialysis independence at 90 days. Rather mortality was increased over the study and 2 years of follow-up in the HCO-HD group, which may have been driven by the increased incidence of serious adverse effects in this group.

## Potential Limitations and Drawbacks of Extracorporeal FLC Removal

The first limitation of this strategy has to do with the efficacy of HCO-HD in reducing the total FLC load. This is because while extracorporeal techniques are highly effective in clearing FLCs from the intravascular space, this space represents only 15%–20% of the volume of distribution of the FLCs. Consequently, despite ≥70% reduction in serum FLCs during each HCO-HD session, it only accounts for a 15%–20% reduction of the total FLC load, when considering a 2-compartment model.^13^ The remaining FLCs will redistribute and may have contributed to the lack of reduction in FLCs seen after the first few days in the EuLITE study. A second concern that has been postulated is that decreasing FLCs with HCO-HD may decrease the effectiveness of proteasome inhibitors. The reason for this is that multiple myeloma cells require an enhanced capacity to handle the increased unfolded proteins, resulting from the increased production of immunoglobulins and FLCs. This function is handled by the proteasome *via* a process called the unfolded protein response (UPR), which protects these cells from accumulating unfolded and misfolded proteins and subsequent cellular damage. If this process is delayed or overwhelmed, terminal activation of this process occurs, resulting in cell cycle arrest and apoptosis. The increased basal activity of the UPR in multiple myeloma cells makes them susceptible to terminal activation of the UPR, which is a predominant mechanism by which proteasome inhibitors work.^16,17^ Because the amount of immunoglobulins retained within the multiple myeloma cells correlates with their sensitivity to proteasome inhibitors, it has been speculated that rapid extracorporeal clearance of FLCs has the potential to decrease their responsiveness to these agents. While this is speculative, it is interesting to point out that the EuLITE study found that patients subjected to HCO-HD had lower chemotherapy response rates than those who received conventional HD (42% vs 68%). A final potential disadvantage is that hematologists rely on sFLC levels to assess the response to chemotherapy. Thus, lowering sFLC levels after HCO-HD may delay the detection of treatment failure and consequently treatment modification.

Multiple myeloma remains a common and frequently lethal disease. Despite the advances in effective chemotherapy, the 5-year mortality remains approximately 50%, which is largely driven by FLC-mediated end-organ damage. Hence, removing sFLC *via* extracorporeal therapies makes much sense from the pathophysiologic perspective. This rationale was particularly valid when older chemotherapy regimens were used because they caused slow decreases in sFLCs, but more questionable in the era of proteasome inhibitor-based chemotherapy that decrease sFLCs more rapidly. Indeed, the best available randomized trials have failed to demonstrate a benefit in hard outcomes. Thus, although beneficial effects of these strategies may exist in select patients, the evidence to date does not warrant the addition of TPE or HCO-HD to modern chemotherapy for most patients.
